# The relationship between parental education and children’s schooling in a time of economic turmoil: The case of East Zimbabwe, 2001 to 2011

**DOI:** 10.1016/j.ijedudev.2016.09.003

**Published:** 2016-11

**Authors:** Erica Pufall, Jeffrey W. Eaton, Constance Nyamukapa, Nadine Schur, Albert Takaruza, Simon Gregson

**Affiliations:** a Department of Infectious Disease Epidemiology, Imperial College London, St. Mary’s Campus, Norfolk Place, London, W2 1PG, UK; b Biomedical Research & Training Institute, No. 10 Seagrave Road, Avondale, Harare, Zimbabwe

**Keywords:** International education, Development, Economic factors, Academic achievement, Zimbabwe

## Abstract

Using data collected from 1998 to 2011 in a general population cohort study in eastern Zimbabwe, we describe education trends and the relationship between parental education and children’s schooling during the Zimbabwean economic collapse of the 2000s. During this period, the previously-rising trend in education stalled, with girls suffering disproportionately; however, female enrolment increased as the economy began to recover. Throughout the period, children with more educated parents continued to have better outcomes such that, at the population level, an underlying increase in the proportion of children with more educated parents may have helped to maintain the upwards education trend.

## Highlights

• We model education trends during the economic turmoil in Zimbabwe.
• During the economic collapse, female education decreased more than male’s.
• Children with more educated parents continued to have better outcomes.
• Despite the collapse, parental and child education has increased over time.
• Increasing proportions of educated parents may have helped maintain education.

## 1 Introduction

Zimbabwe experienced a major economic crisis during the 2000s, from which it is still recovering. The Zimbabwean economy began to decline in the late 1990s, particularly following the ending of the World Bank/IMF supported Economic Structural Adjustment Programme. However, the land redistribution programme, starting in 2000, is viewed as the main catalyst for the economic and social crisis that would define much of the 2000s in Zimbabwe (Koech, 2012). Hyperinflation peaked at almost 500 billion percent in December 2008 (Koech, 2012, Chimhowu, 2009), before the adoption of a multi-currency system (primarily utilising the US dollar) in early 2009.

**Fig. 1:**
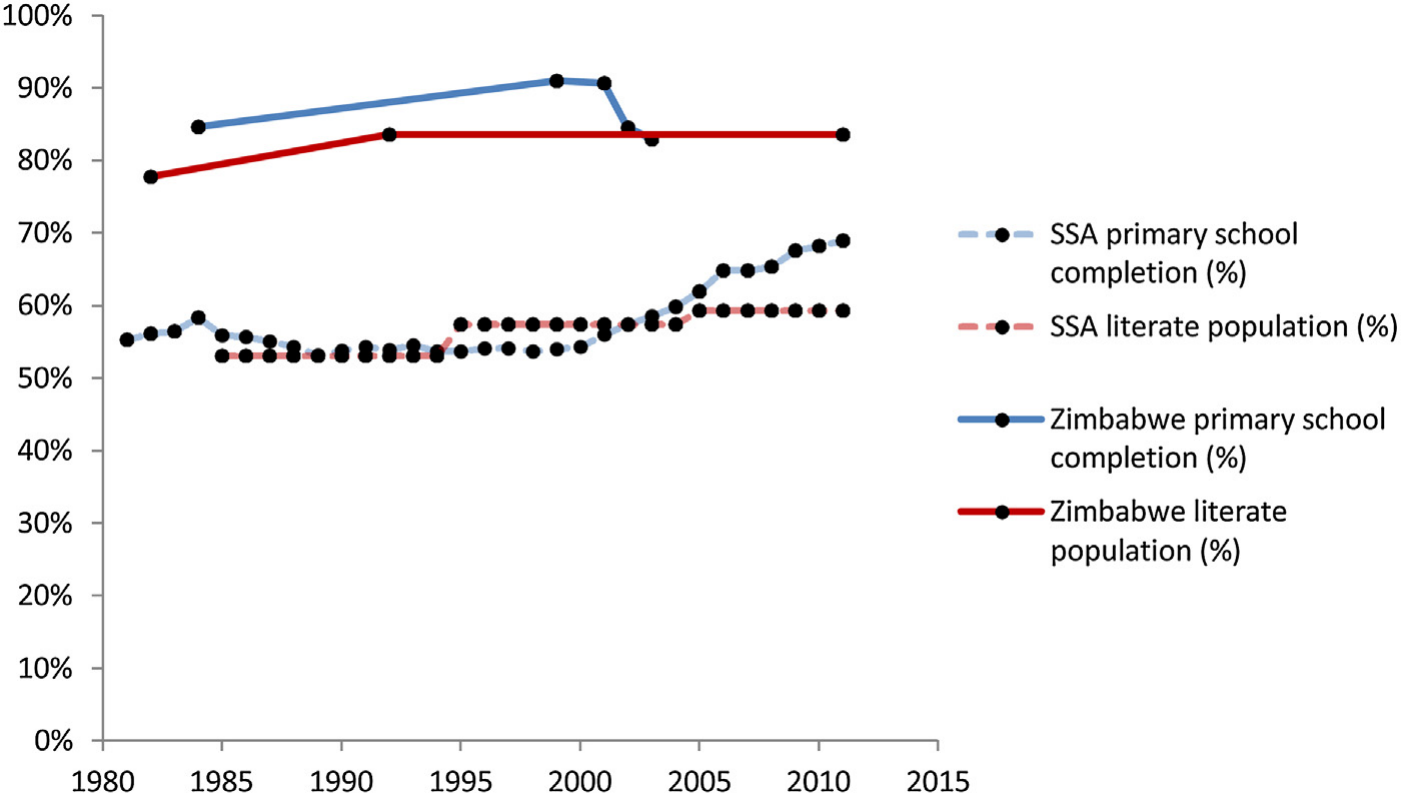
Comparison of primary school completion rates and the proportion of adults (aged 15+) who are literate in Zimbabwe and SSA over time.

Source: The World Bank: Education Statistics: UNESCO Institute for Statistics (World Bank Edstats, 2014).

This crisis was not limited to the financial sector, with news reports suggesting that the education sector, among many others, suffered during this time (Raath, 2008). Contemporary Zimbabwe has some of the highest levels of primary school completion and adult literacy among countries in sub-Saharan Africa (SSA)^1^ (see Fig. 1 based on World Bank Education Statistics) (World Bank Edstats, 2014). This resulted from the implementation of mandatory free primary education after independence in 1980 (Kanyongo, 2005), with universal primary education being achieved in the late 1980s (Chimhowu, 2009). However, primary school fees were re-introduced in 1991 and have risen steadily since then (Kanyongo, 2005). These rising school fees have affected the education system together with macro-economic and social shocks. Declines in productivity led to falls in employment and disposable income, which, coupled with rising primary school fees, meant that many families—particularly those in rural areas—were either unable to afford school fees, or needed their children to contribute to income generation, which forced children to drop out of school (Kiernan, 2008, Koech, 2012). In the 2000s, over 30% of all children who enrolled in primary school dropped out before finishing their final year and summative high school exam (‘O’ level) pass rates fell to just 11% (Chung, 2008). The education sector in Zimbabwe has started to recover since the adoption of the multi-currency system, with schools reopening and large investments being made. Other challenges still face the education system, however, including the lower proportion of females receiving education as compared to males (Koech, 2012). Moreover, rising levels of orphanhood, children caring for sick parents, and other vulnerabilities linked to the HIV epidemic have posed challenges to sustaining and improving education levels in the population (Birdthistle et al., 2009, Nyamukapa and Gregson, 2005, Case et al., 2004, Kembo, 2010, Pufall et al., 2014).

Given Zimbabwe’s education history and the number of national-level events that have influenced the education system, it provides a good setting in which to study education trends during a time of economic turmoil. Previous work in Indonesia has suggested that education may be negatively impacted by economic turmoil, with households reducing their expenditure on health and education by 40% during the late 1990s (Frankenberg et al., 2002), but the associations between macro-economic events and education outcomes have not previously been studied in Sub-Saharan Africa.

Although macro-economic and social changes can influence education, evidence suggests that micro-level determinants can moderate the effects. In other settings, better educated parents have been found to be more likely to educate their children. Parental literacy, years of schooling, and education level are correlated with child education measures in developed countries (Benjamin, 1993, Dickson et al., 2013, Dubow et al., 2009, Chevalier, 2004), with paternal education suggested to be more influential than maternal education in West Africa (Ermisch and Pronzato, 2010).

In this paper, we use data from eastern Zimbabwe to examine education trends during a time of economic decline and crisis, and whether children with more-educated parents are less likely to experience detrimental effects to their education during times of widespread economic hardship. We hypothesise that, at the population level, long-term increases in parents’ education can help to sustain high overall levels of school education during periods of economic decline.

## 2 Data and methods

### 2.1 Study population and data collection

We analysed data from a population-based, open cohort study in 12 geographically distinct study sites in Manicaland province eastern Zimbabwe, which are representative of the Manicaland population (4 subsistence farming areas; 4 large-scale commercial estates; 2 small towns; and 2 roadside settlements) (Gregson et al., 2006, Gregson et al., 2002, Lopman et al., 2008). Manicaland is a primarily rural province and, as rural areas were more likely to suffer during the economic crisis (Kiernan, 2008, Koech, 2012), provides an ideal location to examine education trends during this period.

Population surveillance data were collected during five survey rounds occurring approximately every 2–3 years from 1998 through 2011. Each round of the survey involves a census of all households in the 12 study sites, followed by interviews with individual household members aged 15–54 years. Prior to round three, data were only collected in males aged 17–54 and in females aged 15–44. The main focus of the survey is on trends and risk factors for HIV infection and AIDS mortality (Gregson et al., 2006), but the questionnaires also include questions about education, employment, household assets, and socioeconomic status, enabling investigation of trends in education indicators during the economic collapse.

The survey rounds correspond broadly to the different stages of the economic collapse: Round 1 (1998–2000) corresponds to the period before the worst of the turmoil; round 2 (2001–2003) corresponds to the time of the land redistribution programme and the start of the economic collapse; round 3 (2003–2005) corresponds to the period of rapidly accelerating inflation and growing shortages; round 4 (2006–2008) corresponds to the period of hyperinflation, culminating in economic collapse; and round 5 (2009–2011) corresponds to the adoption of a multi-currency system and the beginning of the economic recovery. Although these are not perfect proxies for stages in the Zimbabwean economic collapse, they do allow us to assess whether education levels changed over time in the face of a rapidly evolving national economy.

Parental data, including information on education, were collected in the Manicaland survey and linked to their children aged 24 and under from round 2 (2001–2003) onwards based on a household roster and, for mothers, confirmed through fertility histories and children’s reports on their biological mothers. Because of this, although education trends were examined overall from 1998 to 2011, but the associations between parental and child education were only tested from 2001 onwards.

Ethical approval for the Manicaland HIV/STD Prevention Project was obtained from the Research Council of Zimbabwe (Number 02187), the Biomedical Research and Training Institute Zimbabwe's institutional review board (Number AP6/97), and the Imperial College London Research Ethics Committee (Number ICREC 9_3_13). Written informed consent was obtained prior to survey participation. Participants were informed that, at any point, they could refuse to answer a question or decline to continue the interview.

### 2.2 Education measures

Six measures of education were examined in the study: enrolment in school (ages 15–16 and ages 17–18), correct grade-for-age (a measure of progression through school), primary school completion, having completed at least one year of secondary or higher education, literacy, and having five or more ‘O’ level passes.

These indicators were selected to provide a mix of process (including educational quality) and outcome (including educational attainment) indicators (e.g. grade-for-age vs. five ‘O’ level passes), and to include indicators that might be expected to be more and less sensitive to the effects of short-term socio-economic shocks (e.g. five ‘O’ level passes at age 16 vs. literacy). School enrolment at ages 15–16 and ages 17–18 were both included as many children leave school after taking ‘O’ levels after the age of 16. Both primary school completion and having at least some secondary schooling were included as, in Zimbabwe, it is very common to complete primary school, but secondary school attendance is not compulsory and far less common. ‘O’ level passes were included because having at least 5 ‘O’ level passes is the minimum requirement for formal sector employment in Zimbabwe.

Respondents were taken to be in the correct grade for their age if they were less than two years behind in school (Miller, 2006). This leeway was given to account for the differing ages when children start school (*i.e.* either age 6 or 7). In the survey design, literacy was assumed in those individuals who had completed at least some secondary schooling, whilst those with no secondary schooling were coded as literate if they verified that they could read a newspaper or short letter in either Shona or English. No data on ‘O’ level passes were collected in the first round of the survey so the indicator on having five or more ‘O’ levels was examined from round two onwards. Parental education measures considered were maternal and paternal literacy and secondary or higher education, and were defined as described above.

### 2.3 Data analysis

All analyses were stratified by gender. We used univariable logistic regression to estimate temporal trends in school enrolment, progression in school, literacy, primary school completion, and ‘O’ level passes, with individual random effects to account for participants present in multiple rounds. Trends for school enrolment, progression in school, and literacy were smoothed using quadratic B-spline functions with knots set at 1998, mid-2004, and 2011. Trends in primary school completion were assessed based on the year an individual turned six (and should therefore have started primary school), with knots at 1960, 1975, 1990, and 2002. Trends in ‘O’ level passes were assessed based on the year an individual turned 16, with knots at 1970, 1985, 2000, and 2011. Trends in the relationships between maternal and paternal education levels and child education levels, as well as in parental education levels on their own, were assessed as above, with knots set at 2001, 2006, and 2011. Trends in parental education levels were measured to determine if the proportion of educated parents rose over the study period. Additional knots were tested for all regressions and found not to influence the trends.

Pooled data over survey rounds 2 through 5 were analysed with logistic regression to assess the association between parental education and child education outcomes, with individual random effects for repeated measurements. Parental literacy and secondary or greater education were assessed as predictors for child’s school enrolment (ages 15–18), being in the correct grade-for-age (ages 15–18), primary school completion (ages 15–24), literacy (ages 15–24), and having five or more ‘O’ level passes (ages 16–24). All regressions were adjusted for linear child age, community type (town, estate, roadside settlement or subsistence farming), socio-economic status (SES, measured using a previously described asset index (Lopman et al., 2007)), and survey round. The same regressions were estimated including an interaction term between survey round and the parental education measure to determine if the effect of parental education changed over time. This assessed whether parental education provided additional protection during the economic instability. The first survey round (1998–2000) was excluded from these analyses because parental data were not linked to child data in this round. Analyses were conducted using Stata/SE 12.1

## 3 Results

### 3.1 Education trends in Manicaland, Zimbabwe

**Fig. 2:**
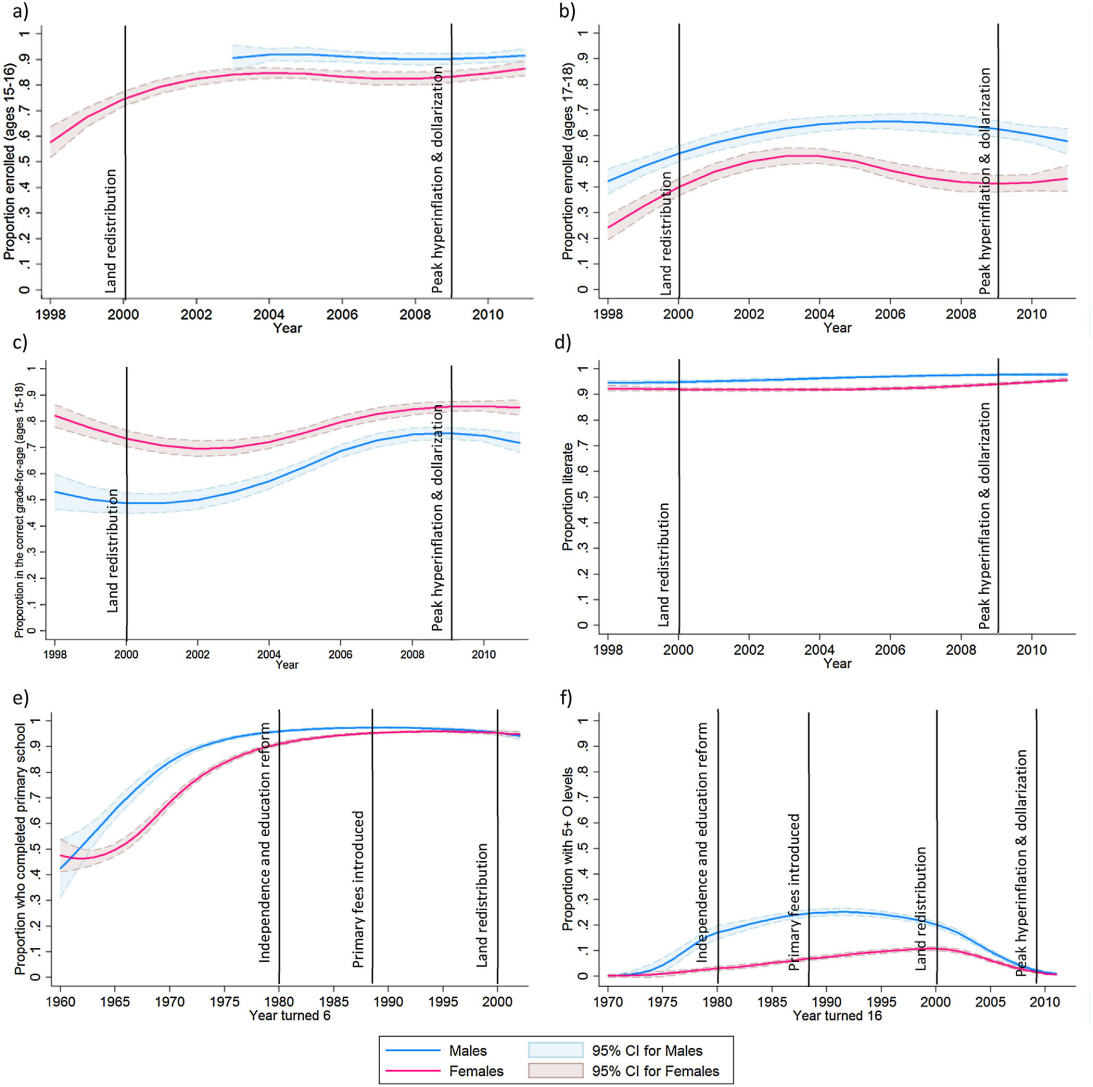
Proportion of males and females a) aged 15–16 enrolled in school; b) aged 17–18 enrolled in school; c) aged 15–18 in the correct grade-for-age; d) who are literate; e) who completed primary school (based on the year they turned 6); and f) who have at least five O level passes (based on the year they turned 16), over time. Ages included are 15–54 unless otherwise noted. Labelled black lines indicate major events in Zimbabwe.

Table 1 describes participant characteristics over the five rounds of the survey. Demographically, the age and gender distribution remained similar across survey rounds, although the percentage of people living in commercial estates has decreased from the earlier rounds. The increase in the percent of children who could be linked to their parents between rounds two and three reflects a change in sampling strategy within households (in round two, we selected (at random) one member of each couple, whereas from round three onwards, all adults aged 15–54 were eligible). Education levels have generally increased over time for both males and females (Table 1 & Fig. 2), although males outperformed females for most education indicators, with the exception of being in the correct grade-for-age (Fig. 2c). Vertical lines on the graphs demarcate major events in Zimbabwe. As expected, more sensitive access-based education indicators, such as school enrolment and having five or more ‘O’ levels at age 16 (Fig. 2a–c), were more volatile than less sensitive attainment-based indicators such as literacy and primary school completion that accumulate over time (Fig. 2d and e). Notably, female access to education was more liable to decrease than male’s, with enrolment and ‘O’ level pass rates dropping more sharply during the socio-economic turmoil of the early- to mid- 2000s. The proportion of people with five or more ‘O’ level passes changed dramatically over time: steadily increasing from 1980 onwards then dropping off starting with the land redistribution in the early 2000s (Fig. 2f). To allow for delays in students achieving five or more ‘O’ levels, we also produced estimates based on the year individuals turned 18, and found a similar decrease in the 2000s (Fig. S1 in Supplementary material), indicating that the reduction was not explained by censoring of delayed ‘O’ level passes in more recent cohorts.

**Fig. 3:**
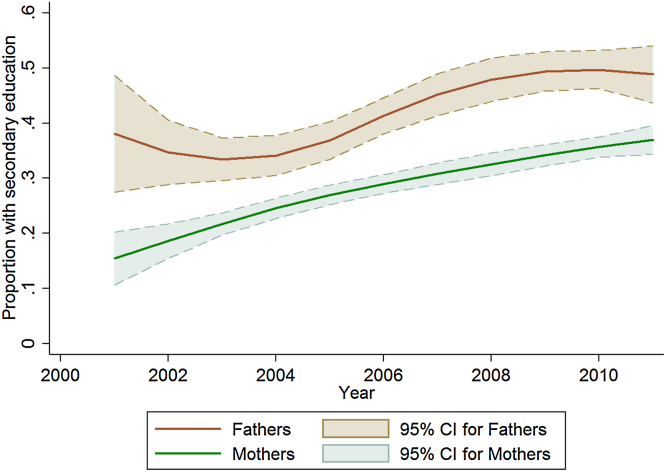
Changes over time in the proportions of identified biological mothers and fathers of youth aged 24 and under who have at least some secondary education.

Education levels among parents increased over the 2000s, as the cohort who experienced the education improvements achieved during the 1980s increasingly entered parenting ages (Fig. 3). The proportion of children and young people under age 25 whose mothers had secondary education roughly doubled between 2001 and 2011, while the proportion with more educated fathers increased by a quarter.

### 3.2 Association between parental and child education

**Fig. 4:**
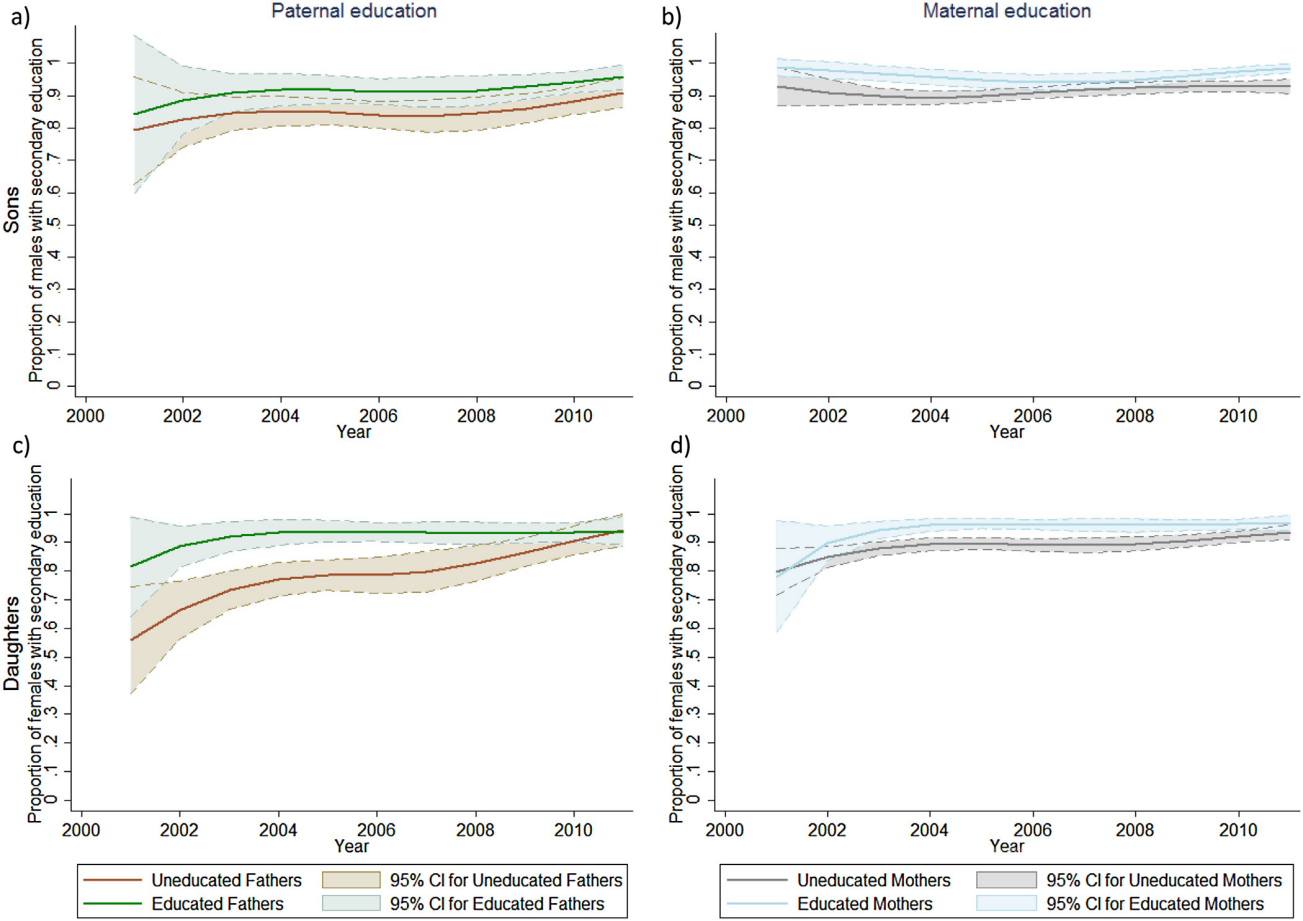
Relationship between paternal (a & c) and maternal (b & d) secondary education levels and the education levels of their sons (a & b) and daughters (c & d) aged 15–24 years, over time.

Table 2 describes the association between parents’ education and their children’s education outcomes. Results are for children ages 15–24 over survey rounds 2–5. Maternal literacy and secondary education were consistently associated with better education outcomes in male and female children. Exceptions were lack of association between maternal literacy and male children being in the correct grade-for-age, and no association between maternal secondary education and child literacy. Paternal literacy was associated with primary school completion in males and females, and paternal secondary education was only associated with primary school completion in females. Whilst mothers’ education shows more statistically significant differences (Table 2), father’s education often makes a bigger absolute difference, particularly for girls before 2009 (Fig. 4c).

To investigate potential bias due to differences between linked and unlinked children, we tested for differences in demographic and education characteristics between youth who were and were not linked to one or both of their parents and found no differences between them (all p > 0.05).

### 3.3 Changes in the relationship between parents’ and children’s education over time

Trends in associations between parental and child education levels (Fig. 4) show that higher levels of both maternal and paternal education have consistent associations with the education level of their daughters over time, but this is not always the case for their sons.

**Fig. 5:**
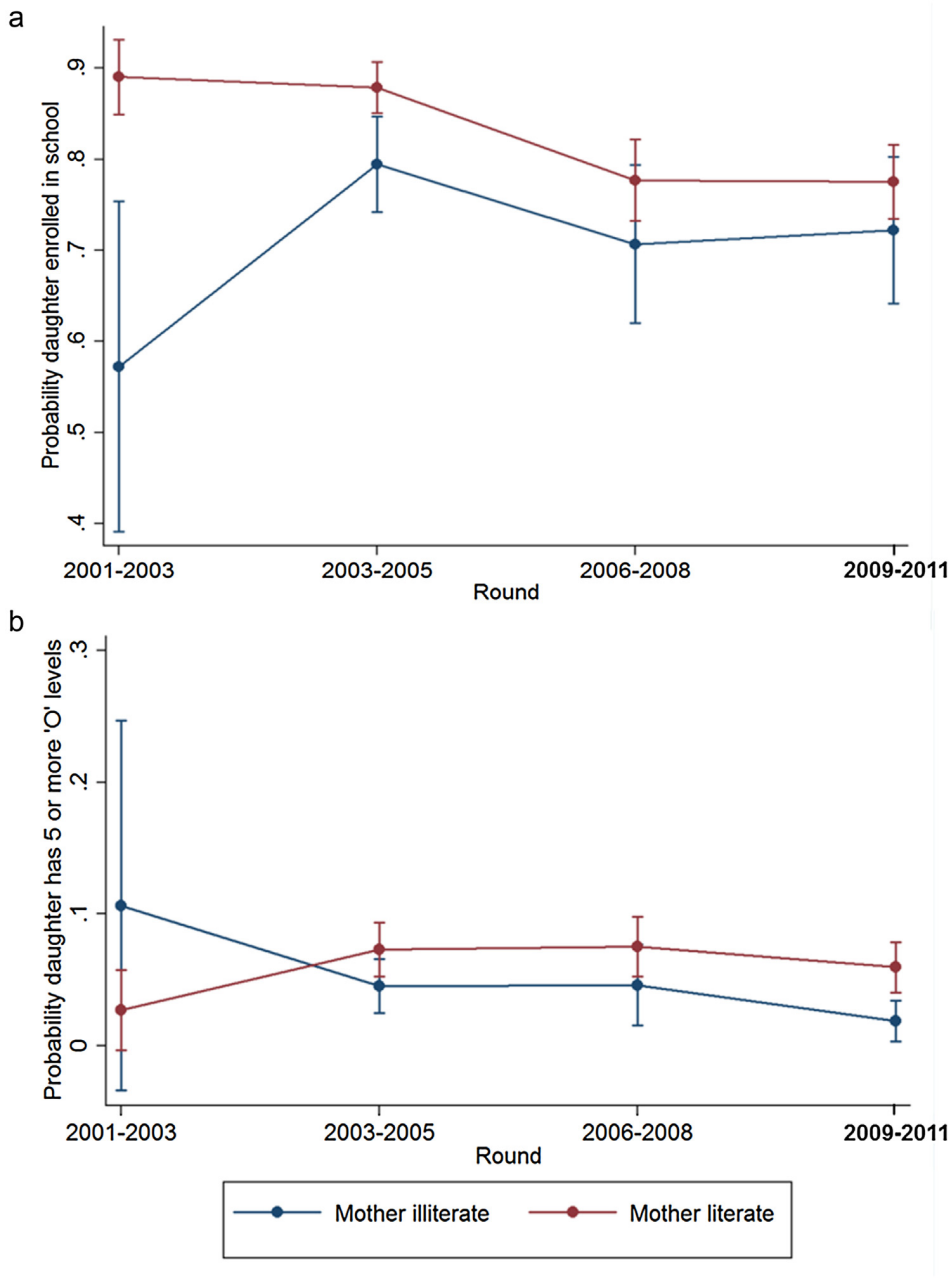
Relationship between maternal literacy and the enrolment (a) and ‘O’ level passes (b) of their daughters aged 16 to 24 by survey round.

Associations between parental and child education (Table 2) were estimated including an interaction term between the parental education measure and survey round to determine if these associations changed over time. Interaction terms were statistically significant in just two cases: the associations of maternal literacy with their daughters’ enrolment and ‘O’ level passes. For enrolment, the positive relationship between maternal literacy and their daughters being enrolled in school appeared to dissipate over time, but the reverse was true for ‘O’ level passes, with maternal literacy having a greater association with increased ‘O’ level passes in the later survey rounds (Fig. 5).

## 4 Discussion

### 4.1 Education levels and national events in Zimbabwe

Generally, education levels have increased over time in Manicaland, Zimbabwe. Primary school completion rates were high and increased among those who were eligible to start primary school from the 1960 to 1975 birth cohorts. Education levels may have begun to increase before 1980 because it was more common for people to enter school late and/or to re-enrol in school, especially when free primary education was introduced in 1980. As of 2011, over 90% of adults aged 15 or older had completed primary school, and over 90% of both men and women are able to read.

Overall, the trends we observed in the education indicators suggest that schooling levels were relatively stable during the socio-economic upheavals that occurred during the 2000s. Indeed, despite the economic turmoil, education levels in Zimbabwe continued to be higher than those in many other sub-Saharan African countries. From 2005 to 2011, youth literacy (ages 15–24) was over 90% in Zimbabwe, as compared to just 64% in sub-Saharan Africa (SSA) as a whole (Unesco, 2014). The levels of literacy we found in the study population are close to those cited in a 2006 UNESCO report, which reported a literacy rate of 97.6% in youth aged 15–24 (Aitchison and Rule, 2006) (it is relevant to note a limitation that our survey only actively tested literacy among respondents who did not complete secondary school, assuming literacy among secondary school completers). Primary school enrolment was much higher than average in Manicaland—there was a net enrolment ratio of just 77% in SSA in 2011, while over 99% of primary school-aged children in the Manicaland study were enrolled in school from 2009 to 2011 (Pufall et al., 2014). This is despite rising tuition fees and the economic uncertainty that Zimbabwe has experienced, suggesting that the legacy of “education for all” (Chimhowu, 2009), which began with Independence, continues.

That being said, and although we could not test the effects of macro-level events directly, the previous rising trend in education stalled during the worst of the economic turmoil and during this period, short-term, access-based education indicators (*e.g.* enrolment for girls aged 17–18) dropped substantially. Similarly, the numbers of people with five or more ‘O’ level passes, which increased steadily from 1980 to the mid-late 1990s, dropped-off rapidly during the 2000s. One should be careful in directly attributing this to the economic crisis however, as the fluctuation of ‘O’ level pass rates may also partly reflect patterns of migration, with more educated people leaving the predominantly rural study areas in Manicaland for more urban areas in Zimbabwe, or for other countries (Zanamwe and Devillard, 2009).

Despite the apparent associations between the economic collapse and education levels, we cannot assume, based on our data, that macroeconomic changes have a direct linear effect on individual education outcomes (Conceição et al., 2009). Economic shocks do not necessarily impact everyone in the same way, and coping strategies may differ depending on sources of livelihood (Lerman and Mckernan, 2008). Some households may have savings and investments; others may be getting remittances; while others still may be in the informal economy, with few links to the formal economy (Robertson et al., 2013, Schur et al., 2015, Crea et al., 2013). Moreover, attaining a particular education status (*e.g.* 5 ‘O’ level passes) is a long process and is likely to be related to the education system as a whole, rather than just an economic shock, and, as such, economic turmoil may not effect educational attainment over the same period as the shock, but may instead be subject to a lag (Meng and Gregory, 2002), and can be offset by household assets (Filmer and Pritchett, 1999). Therefore, assuming a direct link and immediate effect between economic changes and individual education may not be possible. Despite these caveats, our data do show that, regardless of the causal reasons, short-term education measures (*i.e.* enrolment and progression in school) decreased in eastern Zimbabwe during the economic crisis.

### 4.2 Parents’ education, the economic crisis, and children’s education

Over time, children with more educated parents were consistently more likely to be educated themselves. Higher parental education was associated with all the measures of child schooling that we investigated, suggesting that parental literacy and secondary education holistically support the education outcomes of children and are not confined to improving just one outcome. This is consistent with previous research that has found that parents’ education is one of the most important determinants of children’s education (Benjamin, 1993, Dickson et al., 2013, Dubow et al., 2009, Chevalier, 2004, Glick and Sahn, 2000).

Although more educated parents continued to afford their children higher levels of education during the worst of the economic crisis, this effect was no greater than before the worst of the crisis (2001–2003) or in the early years of economic recovery (2009–2011). One exception was a positive association between maternal literacy and their daughters having five or more ‘O’ levels. Therefore, although children of more educated parents still had higher levels of education than children from less educated parents during the height of the economic collapse, rising levels of parental education were not sufficient to prevent deteriorations in education outcomes resulting from the turmoil. This is not entirely surprising as, during the worst of the economic crisis, many schools were closed or, if they were open, many teachers did not report for work because they were not being paid or their pay had become worthless (Raath, 2008); these are not things which parents putting a premium on education could offset.

Although more educated parents were unable to provide full protection against the economic crash for their children, neither did the economic and social turbulence erode the effect of parents’ education in buoying up children’s education outcomes. Given that this was the case, our finding that the proportion of children with more educated parents increased greatly over the period of economic decline and collapse (Fig. 3) suggests that, at the population level, rising parental education helped to sustain high levels of school education during this period. As a consequence, we conclude that the relatively high education levels found amongst today’s youth in Zimbabwe partly reflect increases in their parents’ education following the country’s independence in 1980.

### 4.3 The gender gap in education

Although Zimbabwe has made great strides in increasing the education of its population, males have consistently achieved higher education than females, with the exception being female adolescents still in school being more likely to be in the correct grade-for-age. In the 2000s, school enrolment showed a particularly stark divide between the education of males and females. Before then, males and females either had the same levels of enrolment (ages 15–16) or females were rapidly catching up with males (ages 17–18); however, when the first effects of the economic collapse were felt around 2003 (*e.g.* with a declining labour force (Koech, 2012)), female school enrolment dropped, while that of males remained fairly steady. Encouragingly, from 2009 onwards, and in line with the improving economic climate, female enrolment increased and was again nearing the same levels as that of males.

The gender gap, which persisted throughout the study period, is emblematic of a common pattern in SSA (UNICEF, 2014) and the South African Development Community (Aitchison and Rule, 2006). Of the 53 countries with a Gender Parity Index (GPI) below 0.95, 31 are in Africa and six African countries had fewer than 60 girls per 100 boys entering secondary education in 2008 (Sutherland-Addy, 2008). Zimbabwe fared better, with a GPI of 0.89 for secondary education, but that remains below the threshold of 0.95 that is considered to be “on course” to achieving equal education for males and females (Sutherland-Addy, 2008). Data from SSA consistently suggest that girls are less likely to achieve positive educational outcomes than boys (Bicego et al., 2003, Sutherland-Addy, 2008), a finding which was highlighted in a 2007 UNICEF report (UNICEF, 2006). Recent data support this, suggesting that although the gap is narrowing, a divide still exists (UNICEF, 2014). Gender disparities in education have negative implications for health and economic development: less educated women are more likely to die in childbirth, more likely to marry young, more likely to give birth at a young age, more likely to acquire HIV infection at young ages, less likely to participate in politics, and, importantly, less likely to send their own children to school (UNICEF, 2006). A 2009 paper estimated that large gender inequality in education and employment reduced annual growth by as much as 1.7% (Klasena and Lamanna, 2009).

Although we did not examine the causes of the lower education levels of females in this study, a wide range of reasons have been identified previously and include international aid priorities, national economic policies, societal norms (including views that girls should marry early and spend more time on household chores and caring duties than boys), a lack of schools for girls to attend, and family-level economic decisions (UNICEF, 2006, Sutherland-Addy, 2008). Our analysis also found that maternal education was a stronger predictor of child education than paternal education. This further increases the evidence base for education of girls and young women as a long-term priority for development.

### 4.4 The links between SES and education

A potential limitation of this study is the possibility that the higher SES afforded by greater parental education increased child education rather than parents’ education having a direct effect itself. SES is known to be positively associated with both child (Mcloyd, 1989, Mcloyd, 1998, Sirin, 2005, White, 1982) and parental (Dubow et al., 2009) education, but research suggests that parental education remains an independent predictor of child education. In an analysis of data from several large-scale developmental studies, Duncan and Brooks-Gunn (Duncan and Brooks-Gunn, 1997) found maternal education to be linked to children’s intellectual outcomes even after controlling for a variety of other socio-economic indicators. Additionally, Davis-Kean (Davis-Kean, 2005) found direct effects of parental education, but not SES, on children’s standardized achievement scores, while data from a 40 year cohort study showed the positive effects of parental education on child education to be independent of parental SES (Dubow et al., 2009). Our analysis adjusted for SES using an asset-based wealth index, and so we conclude that our findings are consistent with this existing literature: positive associations between parental and child education outcomes were not explained exclusively by household SES, but suggest a true association between parental and child education.

## 5 Conclusion

Despite the economic turmoil, school education increased over time in Zimbabwe and more sensitive education indicators that were lower during the crisis appear to be recovering to pre-economic crisis levels. Encouragingly, during the times of national economic turmoil, children of parents with greater levels of education continued to be more likely to have positive education outcomes. This association is particularly important for females, who were more likely to see their education suffer in times of economic difficulty. With strong links between maternal and child education, there is therefore a strong rationale for public investments in female education: the intergenerational effects of such programmes will lead to a more highly educated population overall. Zimbabwe remains one of the most highly educated countries in Africa and continued investment in the education system will ensure that future generations can reap the benefits of a good education, as their parents have done before them.

## Figures and Tables

**Table 1:** Characteristics of study participants in each round of the survey in Manicaland, Zimbabwe^a^^,^^b^.

	Round 1: 1998–2000 N = 8929 % (95% CI)	Round 2: 2001–2003 N = 5664 % (95% CI)	Round 3: 2003–2005 N = 13,262 % (95% CI)	Round 4: 2006–2008 N = 10,395 % (95% CI)	Round 5: 2009–2011 N = 11,509 % (95% CI)
Demographic characteristics					
Mean age	26.6 (SD: 8.3)	28.0 (SD: 8.4)	26.2 (SD: 8.3)	26.0 (SD: 8.3)	26.6 (SD: 8.5)
Percent female	56.6% (55.6–57.6%)	59.5% (58.2–60.8%)	58.3% (57.5–59.1%)	57.3% (56.4–58.3%)	58.8% (57.9–60.0%)
Site type					
Town	16.4% (15.7–17.2%)	13.8% (13.0–14.7%)	16.2% (15.6–16.8%)	15.8% (15.1–16.5%)	17.7% (17.0–18.4%)
Commercial estate	32.3% (31.3–33.2%)	33.2% (31.9–34.4%)	29.6% (28.8–30.4%)	26.6% (25.7–27.4%)	26.2% (25.4–27.1%)
Roadside trading centre	17.3% (16.5–18.1%)	17.4% (16.4–18.3%)	19.8% (19.1–20.4%)	20.2% (19.4–20.9%)	20.3% (19.6–21.0%)
Subsistence farming	34.0% (33.0–35.0%)	35.7% (34.4–36.9%)	34.4% (33.6–35.3%)	37.5% (36.5–38.4%)	35.8% (34.9–36.6%)
SES^c^					
Poorest quintile	18.4% (17.6%)	N/A	18.9% (18.3–20.0%)	18.4% (17.6–19.1%)	19.6% (18.9–20.4%)
Second quintile	13.4% (12.7–14.1%)	N/A	15.4% (14.7–16.0%)	13.0% (12.3–13.6%)	19.7% (12.2–13.6%)
Middle quintile	24.6% (23.7–25.5)	N/A	20.0% (19.2–20.6%)	22.6% (21.8–23.5%)	16.6% (15.9–17.3%)
Fourth quintile	21.4% (20.6–22.3%)	N/A	22.0% (21.3–22.7%)	22.8% (22.0–23.6%)	15.6% (14.9–16.2%)
Least poor quintile	22.2% (21.3–23.1%)	N/A	23.8% (23.0–24.5%)	23.2% (22.4–24.0%)	28.4% (27.6–29.2%)
Education measures					
Literate	93.5% (93.0–94.1%)	94.5% (93.9–95.1%)	92.8% (92.4–93.3%)	95.4% (95.0–95.8%)	96.1% (96.7–96.4%)
Completed primary school	84.5% (83.7–85.2%)	85.7% (84.8–86.6%)	90.4% (90.0–90.9%)	93.7% (93.1–94.1%)	95.4% (95.0–95.8%)
Secondary or higher education	62.1% (61.1–63.1%)	66.5% (65.3–67.7%)	73.8% (73.0–74.5%)	79.6% (78.8%–80.3%)	81.7% (81.0–82.4%)
5+ O level passes (ages 16–24)^d^	N/A	11.6% (10.8–12.5%)	11.3% (10.8–11.9%)	14.4% (13.7–15.1%)	11.1% (10.5–11.6%)
Enrolled in school (ages 15–18)	43.4% (40.9–45.8%)	73.3% (70.2–76.3%)	71.4% (69.8–73.1%)	70.4% (68.6–72.2%)	71.0% (69.3–72.7%)
Correct grade-for-age (ages 15–18)	69.6% (66.2–73.0%)	62.7% (58.7–66.6%)	64.4% (62.3–66.5%)	79.9% (78.0–81.8%)	78.4% (76.6–80.3%)
Linkages					
Linked to mother (ages 15–24)	N/A	18.3%	35.6%	35.7%	40.9%
Linked to father (ages 15–24)	N/A	5.6%	10.7%	11.1%	12.5%

a Ages 17–54 for males & 15–44 for females.

b Parental data not linked in round 1.

c SES data not collected in round 2.

d Data not collected in round 1.

**Table 2:** Associations between parental education and child education outcomes, aggregated over rounds two to five.

	School enrolment^a^			Correct grade-for-age^a^			Primary completion^b^			Literacy^b^			5 or more ‘O’ levels^c^		
	N	%	AOR (95% CI)^d^	N	%	AOR (95% CI)^d^	N	%	AOR (95% CI)^d^	N	%	AOR (95% CI)^d^	N	%	AOR (95% CI)^d^
Males															
Maternal Literacy															
Illiterate	458	76.4%	1	323	61.3%	1	676	93.8%	1	672	96.7%	1	571	8.8%	1
Literate	1433	85.2%	1.8 (1.2–2.5)^**^	1179	72.9%	1.3 (0.95–1.8)	1867	98.0%	3.0 (1.8–5.1)^***^	1861	99.0%	2.2 (1.1–4.4)^*^	1481	11.0%	1.6 (1.1–2.4)^*^
Paternal Literacy															
Illiterate	67	74.6%	1	49	53.1%	1	94	90.4%	1	94	94.7%	1	79	5.1%	1
Literate	535	84.5%	2.7 (0.48–15)	431	67.7%	1.9 (0.91–4.1)	690	96.5%	2.5 (1.1–5.6)^*^	688	98.3%	3.4 (0.99–12)	544	8.5%	3.6 (0.99–13)
Maternal Education															
Less than secondary	1189	81.1%	1	919	65.6%	1	1659	97.0%	1	1651	98.6%	1	1375	10.1%	1
Secondary or more	595	89.2%	1.7 (1.2–2.5)^**^	518	78.2%	1.6 (1.2–2.2)^**^	730	98.8%	2.9 (1.3–6.8)^*^	728	99.3%	1.5 (0.57–4.1)	554	12.1%	1.6 (1.1–2.3)^*^
Paternal Education															
Less than secondary	335	82.7%	1	261	61.3%	1	457	95.0%	1	456	97.6%	1	371	7.5%	1
Secondary or more	257	85.6%	1.1 (0.34–3.8)	214	71.5%	1.2 (0.77–1.9)	306	97.7%	1.7 (0.75–3.8)	305	99.0%	2.5 (0.65–9.7)	231	9.1%	1.5 (0.83–2.8)
Females															
Maternal Literacy															
Illiterate	456	70.0%	1	300	74.7%	1	619	93.4%	1	614	96.1%	1	480	4.6%	1
Literate	1403	81.6%	1.9 (1.4–2.7)^***^	1099	83.2%	1.7 (1.2–2.5)^**^	1726	98.1%	3.4 (1.7–6.7)^***^	1719	99.1%	4.6 (1.9–11)^**^	1257	7.2%	2.1 (1.3–3.4)^**^
Paternal Literacy															
Illiterate	77	62.3%	1	43	93.0%	1	97	87.6%	1	96	93.8%	1	74	0.0%	1
Literate	500	78.6%	2.4 (0.98–6.0)	371	79.2%	0.53 (0.18–1.5)	602	96.0%	5.5 (1.1–29)^*^	601	98.0%	2.5 (0.94–6.4)	417	6.2%	N/A
Maternal Education															
Less than secondary	1105	77.7%	1	821	79.5%	1	1433	96.8%	1	1426	98.1%	1	1100	6.5%	1
Secondary or more	621	84.4%	1.6 (1.1–2.2)^**^	502	86.7%	1.5 (1.1–2.2)^*^	726	98.5%	1.8 (0.92–3.6)	721	99.3%	2.6 (0.94–7.3)	500	7.6%	1.5 (0.97–2.3)
Paternal Education															
Less than secondary	287	73.2%	1	189	77.8%	1	346	91.6%	1	345	95.9%	1	253	3.2%	1
Secondary or more	268	81.7%	1.8 (0.97–3.3)	215	82.3%	1.2 (0.68–2.2)	325	98.5%	6.8 (1.3–37)^*^	324	98.8%	2.5 (0.81–8.0)	218	8.3%	1.9 (0.92–3.9)

^a^Ages 15–18, ^b^Ages 15–24, ^c^Ages 16–24, ^d^Adjusted for age, SES, round, and community type, with ID as random effect to account for repeat observations. NB: No parental data were collected in round 1. N/A: Insufficient numbers in each category to run analysis.

%: Percent of children with education outcome.

*, **, *** Significant at p < 0.05, <0.01, <0.01.

## Footnotes

1 Abbreviations: SSA: sub-Saharan Africa; SES: socio-economic status.
