# Supplementary material for: The relationship between parental education and children’s schooling in a time of economic turmoil: The case of East Zimbabwe, 2001 to 2011
Source: Int J Educ Dev. 2016 Nov;51:125–34. doi: 10.1016/j.ijedudev.2016.09.003 (PMC5176343; doi:10.1016/j.ijedudev.2016.09.003)
Supplement: Supplementary file 1 [file mmc1.docx]

**
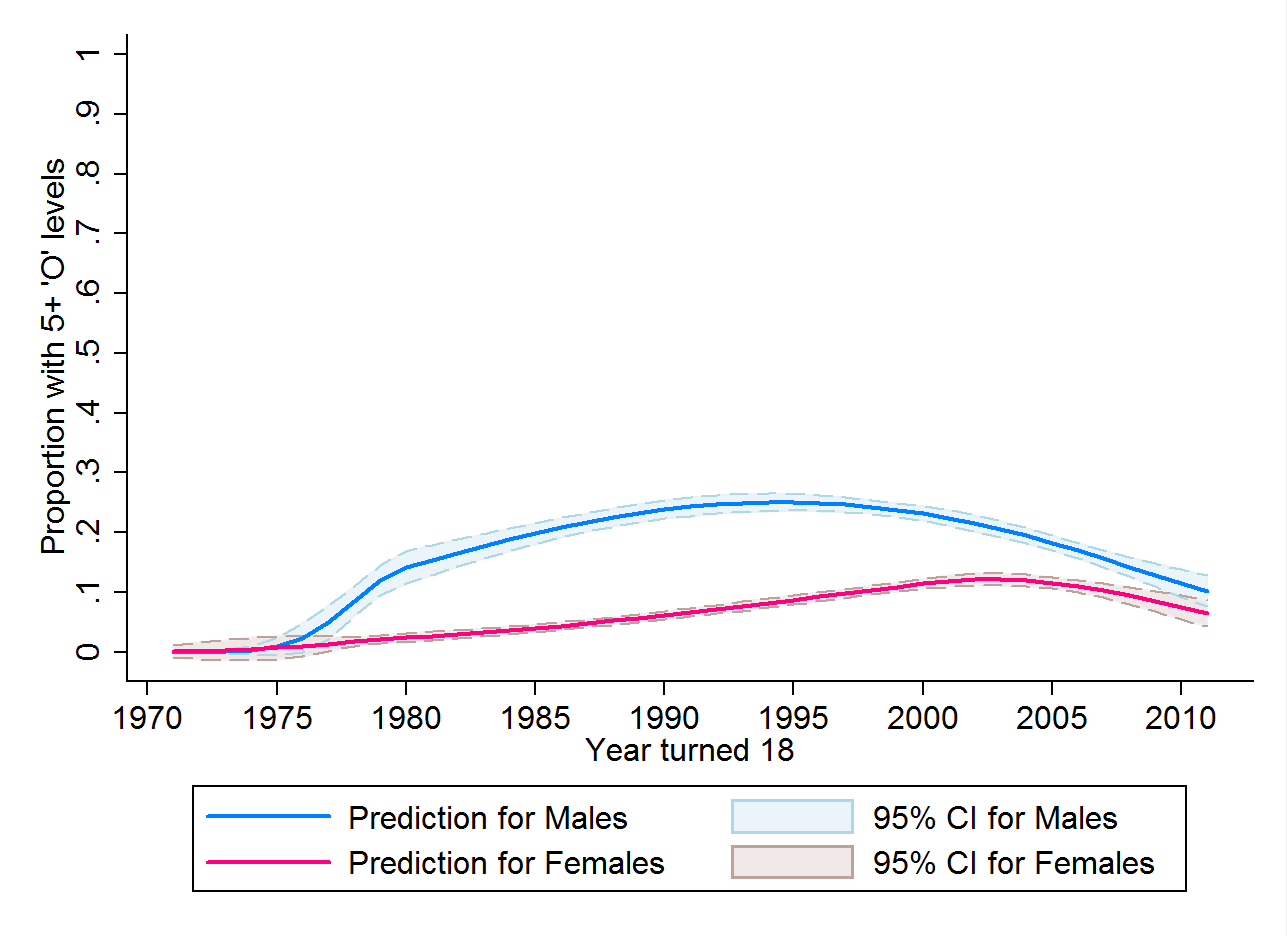
**

Independence and education reform

Primary fees introduced

Land redistribution

Peak hyperinflation & dollarization

**Figure S1** Proportion of males and females aged 18-54 who have at least five O level passes (based on the year they turned 18), over time. Labelled black lines indicate major events in Zimbabwe.
